# Hypothesis testing and sample size considerations for the test-negative design

**DOI:** 10.1186/s12874-024-02277-4

**Published:** 2024-07-16

**Authors:** Yanan Huo, Yang Yang, M. Elizabeth Halloran, Ira M. Longini, Natalie E. Dean

**Affiliations:** 1 Gilead Sciences, Inc, Foster City, CA USA; 2 Department of Statistics, Franklin College of Arts and Sciences, University of Georgia, Athens, GA USA; 3 Department of Biostatistics, University of Washington, Seattle, WA USA; 4 Fred Hutchinson Cancer Center, Seattle, WA USA; 5 Department of Biostatistics, University of Florida, Gainesville, FL USA; 6 Department of Biostatistics & Bioinformatics, Emory University, Atlanta, GA USA

**Keywords:** Test-negative design, Case-control study, Vaccines, Sample size, Score test, Continuity correction

## Abstract

The test-negative design (TND) is an observational study design to evaluate vaccine effectiveness (VE) that enrolls individuals receiving diagnostic testing for a target disease as part of routine care. VE is estimated as one minus the adjusted odds ratio of testing positive versus negative comparing vaccinated and unvaccinated patients. Although the TND is related to case–control studies, it is distinct in that the ratio of test-positive cases to test-negative controls is not typically pre-specified. For both types of studies, sparse cells are common when vaccines are highly effective. We consider the implications of these features on power for the TND. We use simulation studies to explore three hypothesis-testing procedures and associated sample size calculations for case–control and TND studies. These tests, all based on a simple logistic regression model, are a standard Wald test, a continuity-corrected Wald test, and a score test. The Wald test performs poorly in both case–control and TND when VE is high because the number of vaccinated test-positive cases can be low or zero. Continuity corrections help to stabilize the variance but induce bias. We observe superior performance with the score test as the variance is pooled under the null hypothesis of no group differences. We recommend using a score-based approach to design and analyze both case–control and TND. We propose a modification to the TND score sample size to account for additional variability in the ratio of controls over cases. This work enhances our understanding of the data generating mechanism in a test-negative design (TND) and how it is distinct from that of a case-control study due to its passive recruitment of controls.

**Supplementary Information:**

The online version contains supplementary material available at 10.1186/s12874-024-02277-4.

## Introduction

The test-negative design (TND) is an observational vaccine study that is commonly used to monitor the effectiveness of influenza vaccines [1, 2] as well as vaccines targeting rotavirus [3], cholera [4] and COVID-19 [5–7]. It originated from the indirect cohort study to measure pneumococcal vaccine effectiveness (VE) in 1980 [8]. In a typical TND [1], patients seek health care for symptoms of a particular disease, and their specimens are taken for laboratory testing for the vaccine-targeted pathogen. Groups of test-positive cases and test-negative controls are formed according to the test results, analogous to cases and controls in a case–control study. Vaccination history and demographic information of each enrolled individual are recorded. The central assumption of the TND is that the vaccine of interest has no impact on other etiologies of disease [2]. TND can also be used to estimate the relative effectiveness of two vaccines in a direct comparison, or the relative effectiveness of a single vaccine over time by stratifying on time since vaccination. Both test-positive cases and test-negative controls are restricted to people who would seek health care if they experienced symptoms, reducing selection bias due to health-care-seeking behavior [9, 10]. In addition, TND studies are cost-effective, as they require neither prospective follow-up nor active sampling of controls from the community [6]. The studies can be integrated into existing surveillance systems [11].

The TND is commonly analyzed as a case-control study using either logistic [12–15] or conditional logistic regression [16–18]. Covariates often included are age, calendar time, sex, enrollment sites, and comorbidities [5, 6, 19–21]. VE is estimated as one minus the adjusted odds ratio with an associated Wald-based confidence interval and *p*-value [22, 23]. A strength of vaccines is that they can be highly protective, many vaccines against various infectious diseases exhibiting effectiveness above 90%, such as COVID-19 [24] and HPV [25]. As a result, it is not rare to observe low numbers of vaccinated test-positive cases [15]. In these settings, the Wald approach can produce unreliable or even intractable variance estimates. An alternative approach is to add a continuity correction [26, 27] or use exact methods [12, 28–30]. Score testing is another option [31]; when estimating the variance under the null hypothesis of no difference, the groups are pooled, which reduces sparsity. Even if a different null hypothesis is used, the results can still be tractable. However, score tests are not commonly used for TND analyses.

Limited guidance is available on power and sample size calculations for the TND. The TND is fundamentally a passive design, with investigators not having direct control over the number of test-positive cases and test-negative controls observed. Yet power and sample size calculations are useful for determining study feasibility and the broadness of eligibility criteria, planning the number of participating sites, and defining the study’s duration. Investigators may conduct interim analyses of data as part of real-time monitoring, and they may wish to time these only after sufficient data have accrued. The most natural approach for power and sample size calculations is to use case–control equivalents to design the TND. For case control studies, Breslow proposed a sample size corresponding to the Wald test in 1987 [32]. Fleiss modified Breslow’s sample size corresponding to the Wald test adding a continuity correction [33]. The score sample size [21] was developed based on score statistics from a logistic regression. Other sample size methods, such as arcsine transformation sample size [32] and exact test sample sizes [34], were also proposed. A limitation of exact approaches is that a closed-form solution for sample size or power does not exist, although software packages are readily available.

In this article, we examine hypothesis-testing methods, assessing the performance of the Wald test without and with continuity corrections, and a score test based on a case-control study applied to TND data, with a focus on sparse data settings. We also compare the performance of their associated sample size calculations. We explore differences between the case-control and TND studies and identify the added variability relative to the case-control studies due to the random ratio of test-positives to test-negatives in the TND. We proposed a sample size calculation strategy for the TND to mitigate that additional variability.

## Methods

### Sample size methods

We consider three sample size calculation methods corresponding to three different hypothesis tests: a standard Wald test, a Wald test with continuity corrections, and a score test. These are one-sided hypothesis tests for the null and alternative hypotheses of ${H}_{0}:VE\le {1-\theta }_{0}$ and ${H}_{1}:VE>{1-\theta }_{0}, {\theta }_{0}\ge 0$. Data can be summarized in a simple 2 × 2 table with cell counts $a$, $b$, $c$, $d$ as shown in Table 1, and VE is estimated by one minus the odds ratio (*OR*), i.e., $1-\frac{ad}{bc}$. The equivalent null hypothesis is then that the *OR* is greater than or equal to ${\theta }_{0}$, and the equivalent alternative hypothesis is that the *OR* is less than ${\theta }_{0}$, i.e., ${H}_{0}:OR\ge {\theta }_{0}$ and ${H}_{1}:OR<{\theta }_{0}$. Sample size calculations are often based on simplified assumptions, and we discuss the basic scenario without adjusting for confounders here. The approach adjusting for confounders will be similar to a logistic regression with added covariates [35].

**Table 1:** 2 $\times$ 2 contingency table

	**Test positives**	**Test negatives**	**Total number of tests**	**Source population**
**Vaccinated**	a	b		
**Unvaccinated**	c	d		
	${n}_{TP}$	${n}_{TN}$	$n$	$N$

#### Standard Wald test

The standard Wald test is a common test of the log odds ratio, where variance is estimated by the Delta method utilizing the alternative hypothesis. The Wald test statistic ${T}_{W}$ based on the four cell counts in Table 1 is:

$${T}_{W}=\frac{ln \left(\frac{ad}{bc}\right) -ln({\theta }_{0})}{\sqrt{\frac{1}{a}+\frac{1}{b}+\frac{1}{c}+\frac{1}{d}}}$$

With a sufficiently large sample size, this test statistic follows a standard normal distribution, which can be used to derive a corresponding *p*-value. Note that if any of the cell counts are zero, the standard Wald test statistic ${T}_{W}$ is intractable (including 1/0 in the denominator). Similar results occur when fitting a logistic regression via *glm*() in R where the variance is highly inflated resulting in a Wald test statistic near 0.

Corresponding to the standard Wald test, the Fleiss sample size method [33] is widely used in practice for the design of case–control studies. We modify their formula to re-express the sample size in terms of parameters relevant to the TND; these are: VE, the assumed level of vaccine effectiveness; ${p}_{N}$, the expected fraction vaccinated among negative tests, which is a proxy for the vaccination coverage in the source population under the central assumption that the vaccine has no effect on test negative illness; $\left(1-{e}^{-{\Lambda }_{I}\left(\tau \right)}\right)$, the cumulative incidence of test-positive illness in the unvaccinated population, assuming that individuals test positive no more than once during the study period $\tau$ (i.e., gaining immunity after infection with the target pathogen); and ${\Lambda }_{N}(\tau )$, the cumulative hazard of test-negative illness during the study period $\tau$, allowing individuals to repeatedly test negative with different circulating pathogens producing similar symptoms.

From these inputs, we define several related quantities. These are: ${p}_{I}$, the expected fraction vaccinated among positive tests, ${p}_{I}\approx \frac{{p}_{N}(1-VE)}{1-{p}_{N}\times VE}$ (see Supplement); and $\pi$, the expected fraction of test-positive cases amongst all tests (i.e., percent positivity); $\pi$ can be approximated as follows:

$$\pi \approx \frac{\left(1-{p}_{N}\times VE\right)\left(1-{e}^{-{\Lambda }_{I}\left(\tau \right)}\right)}{\left(1-{p}_{N}\times VE\right)\left(1-{e}^{-{\Lambda }_{I}\left(\tau \right)}\right)+{\Lambda }_{N}(\tau )}$$

Alternatively, $\pi$ can be estimated based on historical surveillance data.

The quantity $\pi$ has a parallel to the ratio $k$ of cases to controls that is often specified in case–control studies (e.g. k = 2 for 2:1 controls to cases). In a case–control study with ratio k, the fraction of cases amongst all observations is $\pi =\frac{1}{k+1}$. In case–control studies, this quantity is pre-specified and fixed by design. In a TND, the number of positive tests or negative tests is typically not controlled due to the passive sampling. Then, $\pi$ represents the expected fraction of cases amongst all tests.

The standard Wald sample size with one-sided significance level $\alpha$ and desired power $1-\gamma$ is as follows, adapted for the TND, is as follows:

$${n}_{W}=\frac{{\left\{{Z}_{1-\alpha }\sqrt{\left[\pi {p}_{I}+\left(1-\pi \right){p}_{N}\right]\left[1-\pi {p}_{I}-\left(1-\pi \right){p}_{N}\right]}+{Z}_{1-\gamma }\sqrt{\left(1-\pi \right){p}_{I}\left(1-{p}_{I}\right)+\pi {p}_{N}(1-{p}_{N})}\right\}}^{2}}{\pi {(1-\pi )\left({p}_{I}-{p}_{N}\right)}^{2}}$$

For a TND, ${n}_{W}$ denotes the estimated required total number of tests in the study.

#### Wald test with continuity corrections

To avoid zero cell counts which make the standard Wald test statistic intractable, and to better approximate a normal distribution, a small number $\delta$, referred to as a continuity correction, can be added to each cell count. Various continuity corrections are described in the literature [26, 36, 37]. An example is the Yates’ correction, based on $\delta =0.5$. The continuity-corrected Wald test statistic ${T}_{C}$ is:

$${T}_{C}=\frac{ln \left(\frac{\left(a+\delta \right)\left(d+\delta \right)}{\left(c+\delta \right)\left(d+\delta \right)}\right)-ln({\theta }_{0}) }{\sqrt{\frac{1}{\left(a+\delta \right)}+\frac{1}{\left(b+\delta \right)}+\frac{1}{\left(c+\delta \right)}+\frac{1}{\left(d+\delta \right)}}}$$

For the continuity-corrected Wald test statistic, a corresponding sample size calculation method is the Fleiss sample size with Yates’ correction, a modification of the standard Wald sample size. The corrected sample size ${n}_{C}$ is expressed as a function of ${n}_{W}$, $\pi$, ${p}_{I}$ and ${p}_{N}$:

$${n}_{C}=\frac{{n}_{W}}{4}{\left\{1+\sqrt{1+\frac{2}{\pi {(1-\pi )n}_{W}|{p}_{I}-{p}_{N}|}}\right\}}^{2}$$

#### Score test

The final test considered is a score test based on the likelihood from a simple logistic regression with binary vaccination status [35]. The test statistic ${T}_{s}$ utilizes the estimated variance under ${H}_{0}$:

$${T}_{S}=\frac{{U}_{{H}_{0}}}{\sqrt{\frac{Var({{U}_{H}}_{0})}{n}}}$$

where $n$ is the total number of tests, ${U}_{{H}_{0}}$ is the score under the null and $Var({{U}_{H}}_{0})$ is the variance of ${U}_{{H}_{0}}$ calculated based on the information matrix. For demonstration, when the upper bound of the null set of odds ratio of the hypotheses is 1, i.e.,${\theta }_{0}=1$, the test statistic is simplified as $\frac{{\widehat{p}}_{I}-{\widehat{p}}_{N}}{\frac{{\widehat{\sigma }}_{0}}{\sqrt{n}}}=\frac{\left(ad-bc\right)\sqrt{a+b+c+d}}{\sqrt{(a+c)(b+d)(a+b)(c+d)}}$, where${\widehat{p}}_{I}=\frac{a}{a+c}$, ${\widehat{p}}_{N}=\frac{b}{b+d}$ are empirical proportions vaccinated among test positives and test negatives, and $\frac{{\widehat{\sigma }}_{0}^{2}}{n}=\frac{(a+b)(c+d)}{(a+b+c+d)(a+c)(b+d)}$ is the empirical estimated variance of ${\widehat{p}}_{I}-{\widehat{p}}_{N}$ when${\theta }_{0}=1$. (see supplement for details). Note that the variance in the score test statistic is developed under the null hypothesis. By pooling data from groups under the null hypothesis, the test statistic is tractable even when an individual cell is zero, as long as all margins are non-zero.

For the score test statistic, a corresponding sample size calculation method is as follows:

$${n}_{S}=\frac{{\left({Z}_{1-\gamma }{\sigma }_{1}+{Z}_{1-\alpha }{\sigma }_{0}\right)}^{2}}{{\left({p}_{I}-{p}_{N}\right)}^{2}}$$

where ${\sigma }_{0}^{2}=\frac{1}{\pi (1-\pi )}\left[\pi {p}_{I}+\left(1-\pi \right){p}_{N}\right][\pi \left(1-{p}_{I}\right)+\left(1-\pi \right)\left(1-{p}_{N}\right)]$ and ${\sigma }_{1}^{2} =\frac{1}{\pi (1-\pi )}\frac{{p}_{I}{p}_{N}(1-{p}_{I})(1-{p}_{N})}{\pi {p}_{I}\left(1-{p}_{I}\right)+\left(1-\pi \right){p}_{N}(1-{p}_{N})}$. These terms are related to the variance of the test statistic numerator of the score test statistics ${\widehat{p}}_{I}-{\widehat{p}}_{N}$, where $\frac{{\sigma }_{0}^{2}}{n}$ and $\frac{{\sigma }_{1}^{2}}{n}$ are the assumed variance of the numerator under ${H}_{0}$ and${H}_{1}$, respectively. The variances are derived based on the likelihood of a simple logistic regression (see supplement for details).

### Proposed TND score sample size for high vaccine effectiveness

To account for the additional variability of the fraction of test positives over all tests $\pi$ in the TND, we propose a modification to the case–control score power calculation for high VE. The standard calculation is based on a single assumed fraction $\pi$. We took the summation of the power over all possible values of $\widehat{\pi }=\frac{a+c}{n}$, weighted by a binomial distribution density, since the test-positive infection is independent from the test-negative infection. To calculate the probability of rejection for each value of $\widehat{\pi }$, it is necessary to define two variance terms. The variance of the test statistic numerator ${\widehat{p}}_{I}-{\widehat{p}}_{N}$ under the null is roughly constant across values of $\widehat{\pi }$, which we denote as ${\sigma }_{0}^{2}$. Meanwhile, the variance under the alternative varies. We use a version derived based on a multinomial distribution ${\widetilde{\sigma }}_{1}^{2}(\widehat{\pi }) =\frac{{p}_{I}\left(1-{p}_{I}\right)}{\widehat{\pi }}+\frac{{p}_{N}\left(1-{p}_{N}\right)}{\left(1-\widehat{\pi }\right)}+2{p}_{I}{p}_{N}$ (see supplement).

$$1-\gamma ={\sum }_{k=0}^{n}\text{Pr}\left({T}_{s}<{Z}_{\alpha }|\widehat{\pi }=\frac{k}{n} \right) Pr \left( \widehat{\pi }=\frac{k}{n} \right)={\sum }_{k=0}^{n}Pr \left({T}_{s}<{Z}_{\alpha }|\widehat{\pi }=\frac{k}{n}\right) \left(\begin{array}{c}n\\ k\end{array}\right) {\pi }^{k} {\left(1-\pi \right)}^{n-k}={\sum }_{k=0}^{n}Pr\left(\frac{{\widehat{p}}_{I}-{\widehat{p}}_{N}}{\frac{{\sigma }_{0}}{\sqrt{n}}}<{Z}_{\alpha }|\widehat{\pi }=\frac{k}{n} \right) \left(\genfrac{}{}{0pt}{}{n}{k}\right){\pi }^{k} {\left(1-\pi \right)}^{n-k}={\sum }_{k=0}^{n}Pr\left(\frac{{\widehat{p}}_{I}-{\widehat{p}}_{N}-\left({p}_{I}-{p}_{N}\right)}{\frac{{\widetilde{\sigma }}_{1}\left(\pi \right)}{\sqrt{n}}}<\frac{{Z}_{\alpha }\frac{{\sigma }_{0}}{\sqrt{n}}-\left({p}_{I}-{p}_{N} \right)}{\frac{{\widetilde{\sigma }}_{1}\left(\pi \right)}{\sqrt{n}}}|\widehat{\pi }=\frac{k}{n} \right) \left(\genfrac{}{}{0pt}{}{n}{k}\right){\pi }^{k} {\left(1-\pi \right)}^{n-k}=\sum_{k=0}^{n}\Phi \left(\frac{{Z}_{\alpha }{\sigma }_{0}-\left({p}_{I}-{p}_{N}\right)\sqrt{n}}{{\widetilde{\sigma }}_{1}\left(\widehat{\pi }=\frac{k}{n}\right)}\right)\left(\begin{array}{c}n\\ k\end{array}\right){\pi }^{k} {\left(1-\pi \right)}^{n-k}$$

The proposed score sample size for high VE can be found by grid search from the case-control score sample size until the right-hand side of the equation achieves the desired power.

### Simulations

To compare the case-control studies and TNDs, we performed a simulation study based on the same vaccine effectiveness and same population vaccine coverage. The ratio of cases to controls is fixed by design in the case–control study but variable in the TND, although we fix the expected value of the ratio for the latter so that the studies can be directly compared.

Scenarios we considered across several vaccine effectiveness $VE=$ 30%, 50%, 70%, 90%, 95% with vaccine coverage ${p}_{N}=$ 10%, 30%, 50%, 70%, 90%. Vaccination is assumed to be completed before the study starts. Because vaccination coverage is constant over time, calendar time is not a confounder [38]. $N{p}_{N}$ individuals in the population are randomly selected to be vaccinated and the rest $N(1-{p}_{N})$ remain unvaccinated. An all-or-none vaccine [39] model is adopted. Among vaccinated individuals, $VE\times 100\%$ proportion are randomly selected to be fully protected and the rest are not protected, sharing the same incidence rate with unvaccinated.

To focus on the comparison between the TND and the case–control study, we assume a constant hazard for both test positive and test negative illness, i.e., ${\Lambda }_{I}\left(\tau \right)={\lambda }_{I}\tau$, ${\Lambda }_{N}\left(\tau \right)={\lambda }_{N}\tau$. We generate event times separately for test-positive events and test-negative events. Individuals may test positive only once during the study period, assuming there is some short-term immunity from infection. Their probability of testing positive is either $\left[1-{e}^{-{\Lambda }_{I}\left(\tau \right)}\right]$ or $\left(1-VE\right)\left[1-{e}^{-{\Lambda }_{I}\left(\tau \right)}\right]$, for unvaccinated and vaccinated, respectively. While individuals are not at risk for testing positive again after initially testing positive, they remain in the population and can still test negative. Individuals can test negative many times. Because testing negative is a recurrent event, their expected number of negative tests is ${\Lambda }_{N}(\tau )$; this is not impacted by vaccination status. In the simulation study, we consider $\tau$ as 100 days and constant hazards ${\lambda }_{I}=$ 0.001 $day{s}^{-1}$, ${\lambda }_{N}=$ 0.002 $day{s}^{-1}$. With different combinations of the vaccine effectiveness and vaccine coverages, 1%-10% population will be infected by the test positive pathogen and around 20% population will be infected by test negative pathogens by the end of study. Each individual may receive a maximum of one positive test and up to three negative tests. Any additional tests are disregarded as occurrences of more than three test-negative infections within the same time period are deemed exceptional. Less than 1% of individuals have more than one negative test in the settings considered.

To ensure the study duration is around 100 days, the source population $N$ is calculated based on the expected cell counts. For each combination of vaccine effectiveness $VE$ and vaccine coverage ${p}_{N}$, the unit values of cell counts are calculated using the approach described in Dean et al. [38] (see Supplement for details): $u\left(a\right)=\frac{E\left(a\right)}{N}={p}_{N}\left(1-VE\right)\left[1-{e}^{-{\Lambda }_{I}\left(\tau \right)}\right], u\left(b\right)=\frac{E\left(b\right)}{N}={p}_{N}{\Lambda }_{N}\left(\tau \right), u\left(c\right)=\frac{E\left(c\right)}{N}=\left(1-{p}_{N}\right)\left[1-{e}^{-{\Lambda }_{I}\left(\tau \right)}\right],u\left(d\right)=\frac{E\left(d\right)}{N}=\left(1-{p}_{N}\right){\Lambda }_{N}(\tau )$. The two Wald and the score sample sizes are calculated at 0.025 significance level and 80% desired power based on ${n}_{W}$, ${n}_{C}$ and ${n}_{S}$ formulae. The source population size $N$ then is determined by dividing the preset sample size by the sum of unit cell counts, i.e., $N=\frac{n}{u\left(a\right)+u\left(b\right)+u\left(c\right)+u(d)}$, where $n$ is the calculated sample size. Notice that the source population we consider here is the population who will seek health care and be tested if sick.

The TND data does not require a fixed ratio of test negative controls to test positive cases, so we stop counting events when the number of tests reaches the desired sample size. The case–control data has the fixed ratio $\pi$, so we stop counting test positive events when the number of positive tests reaches $n\pi$. Next, $n\left(1-\pi \right)$ many test negative controls are randomly selected from all test negative events in the population. We also assume 100% sensitivity and 100% specificity of the diagnostic testing. Each scenario runs 100,000 iterations. Simulations are performed using R (R Core Team (2019).

## Results

### Comparison between the test-negative design data and case–control data

**Fig. 1:**
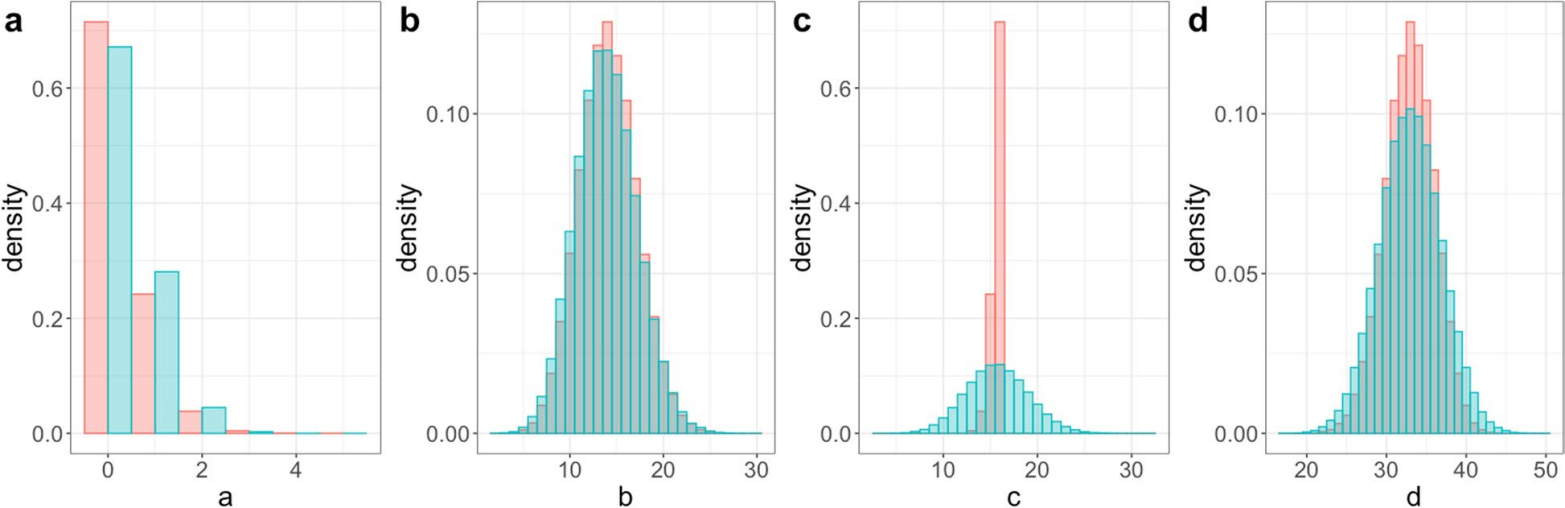
Density of cell counts in the case–control study (red) and test-negative design (green) for 95% VE, 30% vaccine effectiveness ${p}_{N}$ with total sample size of 63. **a** is presented in parallel position and panel (**b**)-(**d**) are presented in identity position

Our simulation results allow us to compare the characteristics of the data generated by a TND and by a comparable case-control study with the same VE, vaccination coverage, and expected ratio of cases to controls. In Fig. 1, we compare the distributions of the four cell counts across the two designs in a setting with 95% VE. The most notable difference was that the distribution of the unvaccinated test positive cases (panel c) had far lower variability in the case-control study. This occurs because the total number of test positives is constrained by design in the case-control study. In contrast, in the TND, only the total number of tests was fixed, yielding greater variability in the individual cell counts. Differences are also visible for panel d, again reflecting the constrained column margins in the case-control study.

Because the standard Wald test is intractable when a zero is present in the cell counts, we compared the frequency of observing zero vaccinated test-positive cases across case–control and TND studies (Table 2). These can be very common for both study types when VE is high and vaccination coverage in the population is low. Overall, we noticed minimal differences in the frequency of zeros between the two designs, although in general more zeros are observed in the case–control study as compared to the TND, particularly when vaccine coverage is low. Thus, both designs are prone to intractability if a standard Wald test is applied.

**Table 2:** Percentage of zero occurred in vaccinated test positive people among 100 K iterations for 95% VE

**Vaccine coverage (**${{\varvec{p}}}_{{\varvec{N}}}$**)**	**10%**	**30%**	**50%**	**70%**	**90%**
**CCT**	68%	67%	64%	57%	22%
**TND**	61%	61%	60%	55%	24%

*CCT* case–control study, *TND* test-negative design

### Adding continuity correction to the Wald test

**Fig. 2:**
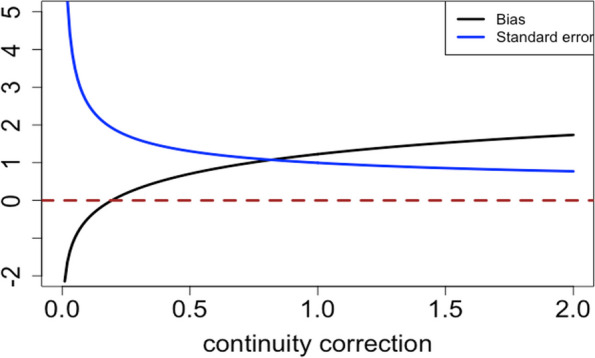
Bias and standard error of log odds ratio for various continuity corrections for 30% vaccine coverage ${p}_{N}$ and 95% VE with naïve Wald sample size 74 in the test-negative design

Moreover, we found that adding continuity corrections to the Wald test stabilized variance but induced bias in the point estimate. In Fig. 2, we scanned the continuity correction from 0 to 2 and evaluated the bias and variance of the log odds ratio. The black line indicates the mean bias of the log odds ratio among 100 k iterations, and the blue line is the standard error of the 100 k log odds ratio estimation. When no continuity correction was added, both bias and variance were intractable since zeros occurred in the denominator. As the continuity correction increased, the estimated variance was stabilized, while the bias increased. Even with the widely used Yates’ correction of adding 0.5 to each cell count, the bias was around 0.5.

### Power performance of the three testing approaches

To broadly compare the three testing approaches and two study designs, we calculated simulated power for vaccination coverage ${p}_{N}$ ranging from 10 to 90%, all assuming 95% VE. For each vaccination coverage level, we calculate the sample size using the Wald formula to achieve 80% power. These sample sizes ranged from *n* = 230 for 10% vaccine coverage to *n* = 37 for 70% vaccine coverage, minimizing at 70% coverage (supplement). We analyze the data using the three tests, substituting a continuity corrected version of the Wald test where the standard Wald test is intractable. The results are shown in Table 3. For both case control studies and TNDs, the two types of Wald tests failed to achieve the desired 80% power, with some exceptions when vaccine coverage was 90%. Vertically comparing the three tests, we found that the score test performed the best across all scenarios. The score test had more stable performance; recall that the score statistic is still tractable when zero cell counts occur. Type I errors for the three tests were well controlled (Table 4). Comparing the case–control and TNDs from equivalent settings, we observed typically lower power for the TND.

**Table 3:** Simulated power under the naïve Wald sample size ${n}_{w}$ for 95% VE at desired power 80% for the three tests

**Tests**	**Studies**	**Vaccine Coverage (**${{\varvec{p}}}_{{\varvec{N}}}$**)**				
		**10%** **(**${{\varvec{n}}}_{{\varvec{w}}}$**=228)**	**30%** **(**${{\varvec{n}}}_{{\varvec{w}}}$**=74)**	**50%** **(**${{\varvec{n}}}_{{\varvec{w}}}$**=45)**	**70%** **(**${{\varvec{n}}}_{{\varvec{w}}}$**=37)**	**90%** **(**${{\varvec{n}}}_{{\varvec{w}}}$**=54)**
**Naïve Wald test**	**CCT**	0.40	0.43	0.49	0.59	0.84
	**TND**	0.42	0.40	0.42	0.46	0.73
**Wald test w. cc**	**CCT**	0.42	0.47	0.61	0.74	0.89
	**TND**	0.37	0.40	0.45	0.52	0.77
**Score test**	**CCT**	0.88	0.86	0.83	0.83	0.86
	**TND**	0.86	0.83	0.79	0.76	0.*82*

*Wald test w. cc* Wald test with continuity correction, *CCT* case–control study, *TND* test-negative design

**Table 4:** Type I errors for the case–control study (CCT) and test negative design (TND) across 10% to 90% vaccine coverage with 100 sample size. The target type I error rate is 0.025. Wald test w. cc: the Wald test adding Yates’ correction

**Tests**		**Vaccine Coverage** (${{\varvec{p}}}_{{\varvec{N}}}$)				
		**10%**	**30%**	**50%**	**70%**	**90%**
**Naïve Wald test**	**CCT**	0.001	0.020	0.025	0.026	0.023
	**TND**	0.015	0.016	0.022	0.022	0.018
**Wald test w. cc**	**CCT**	0.001	0.018	0.025	0.025	0.024
	**TND**	0.001	0.014	0.021	0.022	0.019
**Score test**	**CCT**	0.015	0.023	0.025	0.025	0.028
	**TND**	0.015	0.019	0.023	0.023	0.025

**Fig. 3:**
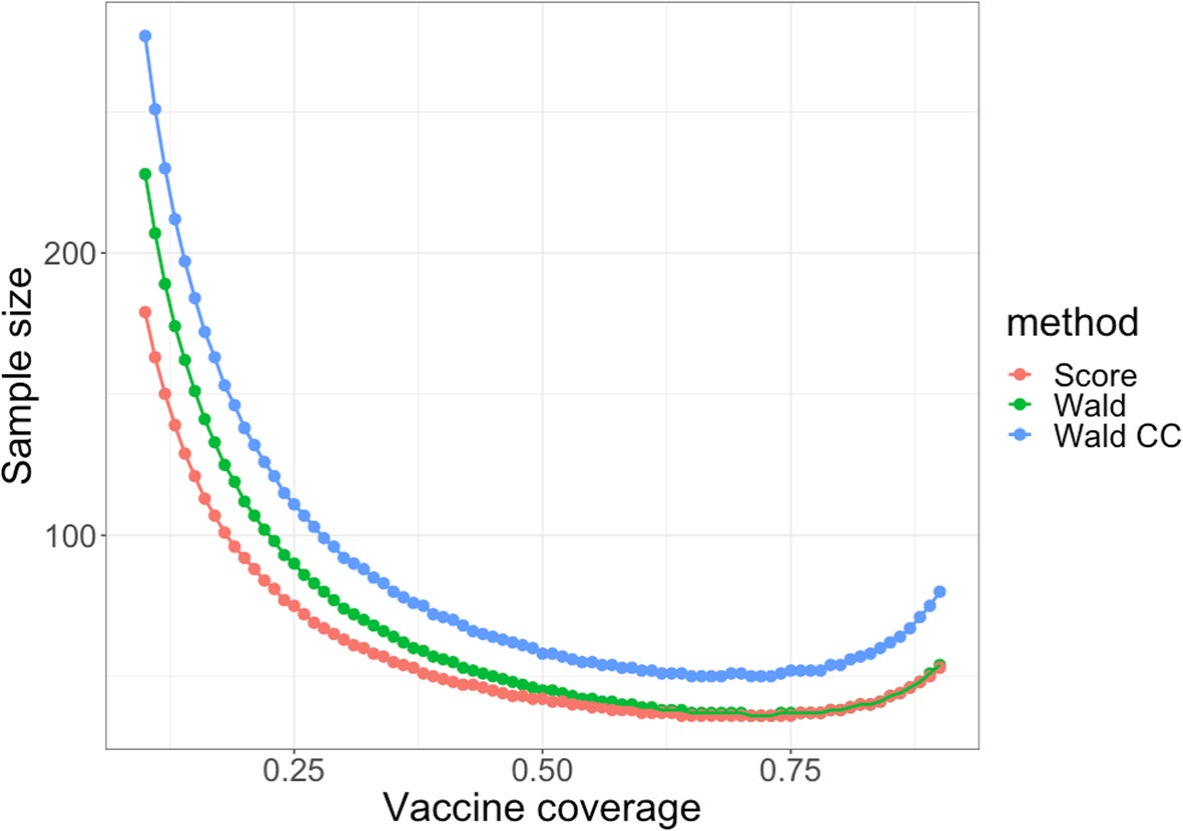
Standard Wald sample size (green), Wald sample size with continuity correction (blue) and score sample size (red) vary with 10%-90% vaccine coverage for 95% vaccine effectiveness

Next, we compared the sample size calculation methods corresponding to each of the three tests. From Fig. 3, we observe that adding the continuity correction increased the Wald sample size by 20% to 50% for 95% VE. The score sample size was the smallest across all scenarios. The standard Wald sample size is similar to the score sample size for 10%-90% vaccine coverage. The required sample size for low vaccine coverage is the largest, while 70% vaccine coverage requires the smallest sample size. As vaccine coverage increases up to 90%, the sample size increases; this reflects more sparsity in the unvaccinated cells in the table.

Next, we considered the simulated power for three scenarios: (i) the standard Wald test but with continuity correction for zero vaccinated test positive along with the standard Wald sample size, (ii) the Wald test with continuity correction along with the standard Wald sample size with continuity correction, and (iii) the score test along with score sample size. The standard Wald test was not evaluated since it is frequently intractable.

**Fig. 4:**
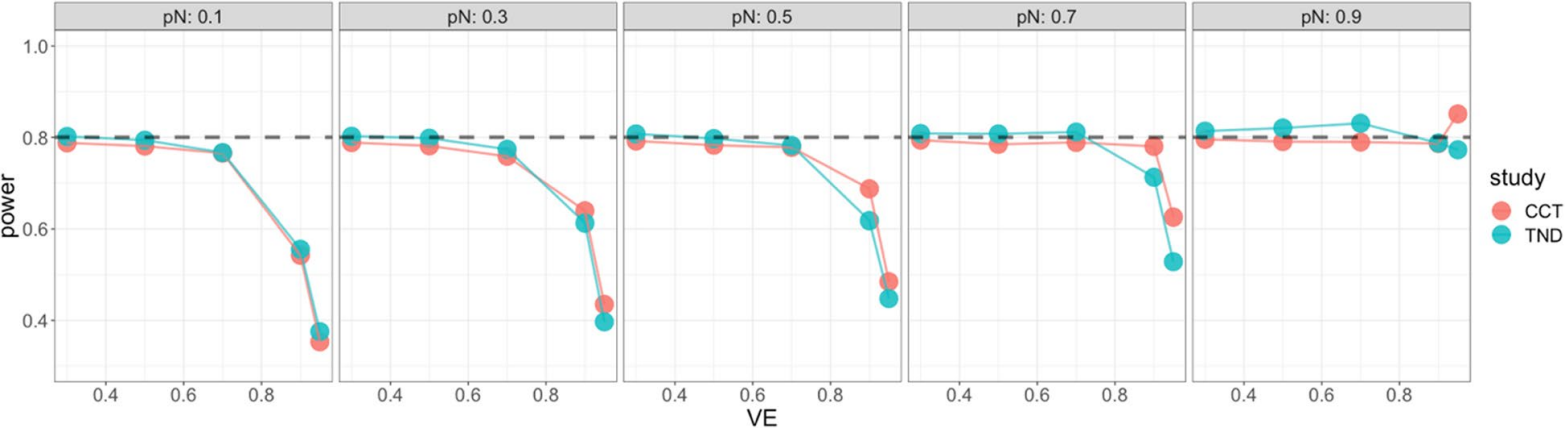
Simulated power for the case-control study (red) and the test-negative design (green): the standard Wald test but with continuity correction for zero vaccinated test positive with standard Wald sample size. x axis: vaccine effectiveness, y axis: simulated power. Vaccine coverage ${p}_{N}$ varies from 10 to 90% for different panels. Desired power is 80%

**Fig. 5:**
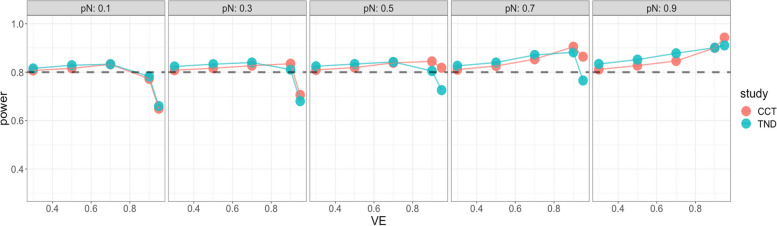
Simulated power of the Wald test with continuity correction with Wald sample size adding continuity correction for case control (red) and test-negative design (green). x axis: vaccine effectiveness, y axis: simulated power. Vaccine coverage ${p}_{N}$ varies from 10 to 90% for different panels. Desired power is 80%

**Fig. 6:**
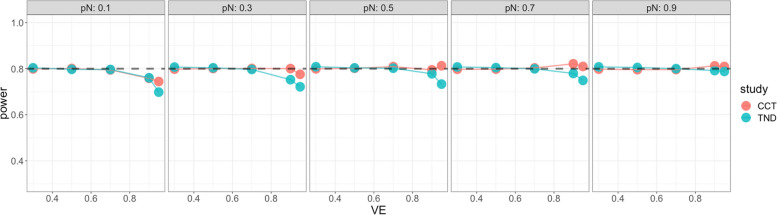
Simulated power of score test with score sample size for case control (red) and test-negative design (green). x axis: vaccine effectiveness, y axis: simulated power. Vaccine coverage ${p}_{N}$ varies from 10 to 90% for different panels. Desired power is 80%

Starting with the standard Wald sample size and test, Fig. 4 shows very low power for both case–control and test-negative design studies when VE is high, especially for low vaccine coverage, indicating insufficient standard Wald sample size. For the continuity corrected Wald sample size and test, Fig. 5 shows low power for both types of studies when VE is high and vaccination coverage is low, but high power (above targeted 80%) when both VE and vaccination coverage are high; this indicates that sample size is insufficient for low vaccine coverage but conservative for high vaccine coverage. For the score sample size and test, Fig. 6 shows that power was maintained around the desired power.

**Fig. 7:**
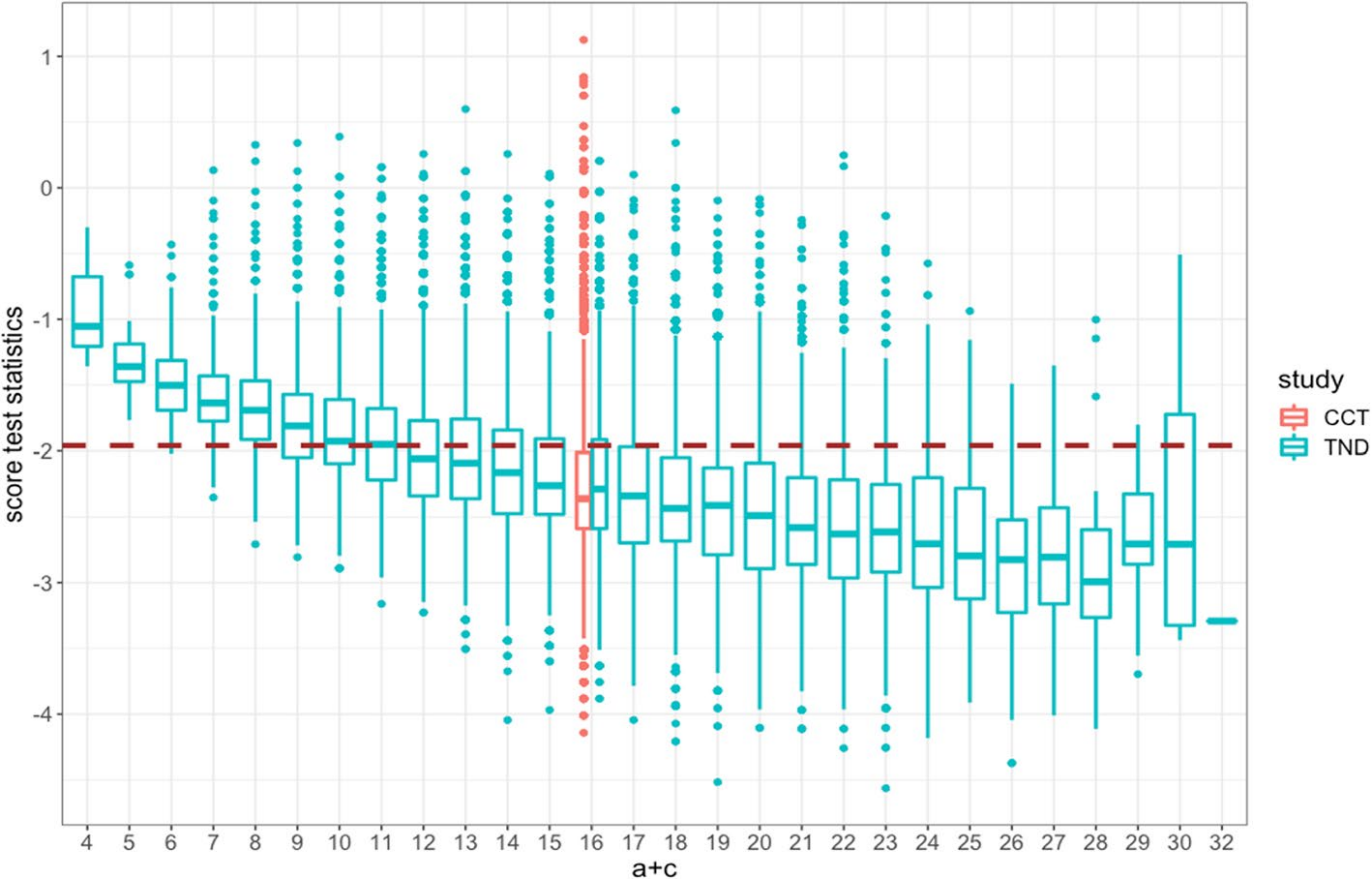
Distribution of score test statistics for 30% vaccine coverage ${p}_{N}$, 95% VE for the case–control study (red) and the test-negative design (green). Brown dashed line indicates the critical value for the test statistic at the 0.025 significance level

In some of the scenarios where VE is high (90% and 95%), we observe lower power for the TND even though power was sufficient for the case-control study. To explore the reason for this discrepancy, we studied the estimated variance of the score as a function of the total number of test-positives (Fig. 7). Recall that the total number of test positives ($a+c$) is fixed by design in the case-control study but varies for the TND. When the total number of test positives in the TND is similar to the fixed value for the case-control study (shown in red), both designs have similar variability in the score test statistics. Yet when the total number of test positives is higher than expected, the TND score statistic has greater variance, and when the total number of test positives is lower than expected, the TND score statistic has lower variance. Thus, there is overall higher variability in the score statistic of the TND than in the case-control study, which is not reflected in the sample size calculations based on the case-control design.

### Proposed TND score sample size and power performance for high vaccine effectiveness

Table 5 illustrates the proposed score sample size and the case-control score sample size for 90% and 95% vaccine effectiveness. The proposed score sample size was relatively larger than the case-control sample size across various vaccine coverage for high VE, since it accounted for the additional variability in the TND.

**Table 5:** Proposed TND score sample size (${n}_{p}$) and the case–control score sample size (${n}_{s}$) for 90% and 95% vaccine effectiveness (VE) across 10%-90% vaccine coverage (${p}_{N}$) with 0.025 type I error and 80% desired power

**VE**	**Vaccine Coverage (**${{\varvec{p}}}_{{\varvec{N}}}$**)**									
	**10%**		**30%**		**50%**		**70%**		**90%**	
	${{\varvec{n}}}_{{\varvec{s}}}$	${{\varvec{n}}}_{{\varvec{p}}}$	${{\varvec{n}}}_{{\varvec{s}}}$	${{\varvec{n}}}_{{\varvec{p}}}$	${{\varvec{n}}}_{{\varvec{s}}}$	${{\varvec{n}}}_{{\varvec{p}}}$	${{\varvec{n}}}_{{\varvec{s}}}$	${{\varvec{n}}}_{{\varvec{p}}}$	${{\varvec{n}}}_{{\varvec{s}}}$	${{\varvec{n}}}_{{\varvec{p}}}$
**90%**	225	268	81	91	55	60	50	54	82	95
**95%**	179	228	63	75	46	46	36	39	53	62

Table 6 shows the simulated power under the proposed sample size improved compared to the case-control score sample size across different vaccine coverages for 90% and 95% VE. The proposed sample size tended to be conservative, especially for low vaccine coverages.

**Table 6:** Simulated power of the score test for the test-negative design under the proposed TND score sample size (${n}_{p}$) and the case–control score sample size (${n}_{s}$) in Table 5. Desired power is 80%

**VE**	**Vaccine Coverage (**${{\varvec{p}}}_{{\varvec{N}}}$**)**									
	**10%**		**30%**		**50%**		**70%**		**90%**	
	${{\varvec{n}}}_{{\varvec{s}}}$	${{\varvec{n}}}_{{\varvec{p}}}$	${{\varvec{n}}}_{{\varvec{s}}}$	${{\varvec{n}}}_{{\varvec{p}}}$	${{\varvec{n}}}_{{\varvec{s}}}$	${{\varvec{n}}}_{{\varvec{p}}}$	${{\varvec{n}}}_{{\varvec{s}}}$	${{\varvec{n}}}_{{\varvec{p}}}$	${{\varvec{n}}}_{{\varvec{s}}}$	${{\varvec{n}}}_{{\varvec{p}}}$
**90%**	0.76	0.88	0.75	0.86	0.78	0.84	0.78	0.83	0.79	0.84
**95%**	0.69	0.90	0.72	0.85	0.73	0.82	0.75	0.82	0.79	0.83

## Discussion

We examined properties of the TND in comparison to a standard case-control study, with a focus on hypothesis testing and sample size calculation. We considered two Wald-based methods and a score-based method. For hypothesis testing, a key disadvantage of the Wald test is that it can be intractable for high VE because of sparsity in the number of vaccinated test positives. Adding continuity corrections to the Wald test enabled estimation but induced bias. For both the TND and case-control study, the score test was more robust across settings, particularly for high VE. Thus, we recommend score-based approaches for testing the vaccine effect in the logistic regression model. The score test can be readily fit using standard statistical software, and it would represent an improvement over Wald-based approaches, which are common in the TND literature [22, 23].

With respect to sample size calculation methods, we recommend a score-based approach adapted from the case–control literature. When accompanied with score-based testing, we found this approach to be the most robust at maintaining the desired power. We detected a slight reduction in power for the score-based sample size in settings with high VE and low vaccination coverage. This reduction in power was more pronounced for the TND when compared to a traditional case–control study. While the ratio of cases to controls is constrained in case–control studies, this ratio is itself a random variable in TNDs. This is due to the TND’s passive sampling scheme, where patient enrollment relies on health-care-seeking behavior and is not controlled by the investigators [10, 40]. With too few test positives captured, the score test statistic is closer to the null value. We proposed a modified score sample size strategy for high vaccine effectiveness to account for the additional variability of this ratio with variance calculated under the multinomial distribution. This approach enhances the power performance but provides conservative sample sizes. This work indicates that sample size calculation methods based on case–control designs have limitations when applied to TNDs and so should not be used uncritically. In this setting, study planning with simulation is another valuable tool.

The additional variability on the column margin in the contingency table results in the TND cell counts followed a multinomial distribution rather than a binomial distribution with one-way variability as in the case–control data. Therefore, the likelihood linked with the logistic regression is not able to fully describe the variance of the vaccine coverage between test positives and test negatives, especially for high vaccine effectiveness and low vaccine coverage (few vaccinated test positives). With the distribution-based variance, the proposed sample size tends to yield power higher than desired. An alternative approach not considered here is to derive a score test sample size from a multinomial distribution linked regression.

The work has several limitations. We considered a simplified scenario with constant vaccine coverage, constant VE, and constant disease hazard over time. We did not consider patterns of health care seeking among the source population. The study population we considered is the population who will seek care if sick. Investigators need to account for the fraction of seeking health care if consider the health-care-seeking behavior varies by vaccination status [38], but the testing strategies and power calculations are similar. We also assumed the diagnostic test has perfect sensitivity and specificity [41]. Furthermore, we do not consider confounders, such as age or risk status that are commonly included in TND analysis. We simplified the scenario to focus attention on sample size calculations, which are frequently conducted using a variety of simplifying assumptions. Nonetheless, we expect the central points about sparsity at high causing a breakdown in the analysis and the role of added variability in the ratio of positives to negatives to carry forward into more complex settings. Other analytical methods, such as exact methods [29, 30] and Bayesian methods [42–44], are also discussed in the literature but not used here. Exact methods are usually applied in the sparse data settings. More importantly, they lack a closed-form solution for sample size calculations, which is our focus. Prior research has also demonstrated that both unconditional and conditional exact test always control the type I error and the unconditional exact test is more powerful at the price of a higher computational burden [29, 30]. Furthermore, while exact methods are useful for 2 × 2 calculations, model-based methods will be required for analyzing test negative data, where adjustment for confounders is required in real-world applications. The continuity-corrected Wald test also has a link to Bayesian methods with the added cell counts akin to a non-informative prior. Considering the distribution of the test-negative design data, the score test approach based on multinomial distribution can be an alternative for the sample size determination. Finally, we have framed the problem as a hypothesis test to assess whether VE > 0% or relative VE > 0% (in the case of a head-to-head comparison or vaccine waning). Investigators may prefer to test a different null hypothesis or seek a desired precision for the point estimate. This would require further modification.

The TND is a relatively new observational study design that is rapidly growing in popularity. Though it is in many ways similar to case–control studies, it has distinct features resulting from how cases and controls are passively sampled [40]. The convenient sampling method induces extra variability on the number of test positives and the number of test negatives. Unlike in a case-control study, the ratio of cases to controls is not governed by the investigator, which can result in imbalances, with many more cases than expected or many more controls. In practice, while at the outset of a TND study, it may be difficult to predict the number of tests that will accrue and their positivity, these approaches can help investigators assess the potential power of their study and can impact planning decisions such as the number of sites to include and patient eligibility criteria. By our examination, we recommend using score test and score sample size under the case–control framework to design the study. Modifications of the score sample size were proposed to account for the additional variability on the ratio of cases over all tests. The work expands our understanding of the data features of the TND relative to a case–control design, bridging gaps in design approaches for the TND.

### Supplementary Information

Supplementary Material 1.

## Data Availability

The research described in this manuscript did not involve the use of any real data or materials.
