# Supplementary material for: Hypothesis testing and sample size considerations for the test-negative design
Source: BMC Med Res Methodol. 2024 Jul 16;24:151. doi: 10.1186/s12874-024-02277-4 (PMC11251325; doi:10.1186/s12874-024-02277-4)
Supplement: Supplementary file 1 — Supplementary Material 1. [file 12874_2024_2277_MOESM1_ESM.docx]

**Hypothesis testing and sample size considerations for the test-negative design**

**Supplement**

Yanan Huo^1^, Yang Yang^2^, M. Elizabeth Halloran^3,4^, Ira M. Longini, Jr.^5^, Natalie E. Dean^6^

*^1^Gilead Sciences, Inc, Foster City, CA, USA*

*^2^Department of Statistics, Franklin College of Arts and Sciences, University of Georgia, Athens, GA, USA*

*^3^Department of Biostatistics, University of Washington, Seattle, WA, USA*

*^4^Fred Hutchinson Cancer Center, Seattle, WA, USA*

*^5^Department of Biostatistics, University of Florida, Gainesville, FL, USA*

*^6^Department of Biostatistics & Bioinformatics, Emory University, Atlanta, GA, USA*

1. **Logistic regression and its likelihood**

The simple logistic regression associated with a test-negative design is

$$logit\left( Pr(Y=1|X) \right)=\beta_{0}+\beta X$$

where$logit\left( Pr(Y=1|X) \right)=\log\left( \frac{\Pr\left( Y=1 | X \right)}{1-\Pr\left( Y=1 | X \right)} \right)$. $Y$ indicates the binary disease status, test positive or test negative; $X$ indicates the binary vaccination status, vaccinated or not; $\beta_{0}$ is interpreted as the log odds of infection against the target pathogen among vaccinated; $\beta$ is interpreted as the log odds ratio of test positives among vaccinated over those among unvaccinated. Because vaccine effectiveness is expressed by $1-OR=1-e^{\beta}$, then $\beta$ is of interest. The hypotheses $H_{0}:VE\leq0$, v.s., $H_{1}:VE>0$ are equivalent to $H_{0}:\beta\geq0$, v.s., $H_{1}:\beta<0$.

The likelihood associated with the logistic regression is

$$L(\beta_{0},\beta)=\pi_{1}^{a}\left( 1-\pi_{1} \right)^{b}\pi_{0}^{c}\left( 1-\pi_{0} \right)^{d}=\left[ \frac{e^{\beta_{0}+\beta}}{1+e^{\beta_{0}+\beta}} \right]^{a}\left[ \frac{1}{1+e^{\beta_{0}+\beta}} \right]^{b}\left[ \frac{e^{\beta}}{1+e^{\beta}} \right]^{c}\left[ \frac{1}{1+e^{\beta}} \right]^{d}$$

where $\pi_{1}=\Pr\left( Y=1 | X=1 \right)=\frac{e^{\beta_{0}+\beta}}{1+e^{\beta_{0}+\beta}}$, $\pi_{0}=\Pr\left( Y=1 | X=0 \right)=\frac{e^{\beta}}{1+e^{\beta}}$. The information matrix is

$I=\left[ \begin{matrix} I_{00} & I_{10} \\ I_{10} & I_{11} \end{matrix} \right]=n\left[ \begin{matrix} \pi_{1}\left( 1-\pi_{1} \right)\bar{p}+\pi_{0}\left( 1-\pi_{0} \right)(1-\bar{p}) & \pi_{1}\left( 1-\pi_{1} \right)\bar{p} \\ \pi_{1}\left( 1-\pi_{1} \right)\bar{p} & \pi_{1}\left( 1-\pi_{1} \right)\bar{p} \end{matrix} \right]$,

where  $\bar{p}=\pi p_{I}+\left( 1-\pi\right)p_{N}$ is the pooled vaccine coverage and $\pi=Pr(Y=1)$ is the pooled probability of testing positive.

1. **Score test statistics with likelihood-based variance**

The score statistics associated with $\beta$ based on the logistic regression is $\sum_{i=1}^{n} X_{i}(I_{\left\{ Y_{i}=1 \right\}}-P(Y_{i}=1|X_{i})).$Under $H_{0}$, the probabilities of being test positive among vaccinated and unvaccinated are the same, then $\Pr\left( Y=1 \right)=Pr(Y=1\left| X=1 \right)=\Pr\left( Y=1 | X=0 \right), i.e, \pi=\pi_{1}=\pi_{0}$. Sequentially, by dividing $n\hat{\pi}(1-\hat{\pi})$, the score statistics can be simplified to $\frac{1}{n\hat{\pi}}\sum_{TP} X_{i}-\frac{1}{n\left( 1-\hat{\pi} \right)}\sum_{TN} X_{i}=\frac{a}{a+c}-\frac{b}{b+d}=\hat{p}_{I}-\hat{p}_{N}$, the empirical vaccine coverage difference between test positives and the test negatives. The information matrix under $H_{0}$ is simplified as

$\tilde{I}=\left[ \begin{matrix} \tilde{I}_{00} & \tilde{I}_{01} \\ \tilde{I}_{10} & \tilde{I}_{11} \end{matrix} \right]=n\left[ \begin{matrix} \pi\left( 1-\pi\right) & \pi\left( 1-\pi\right)\bar{p} \\ \pi\left( 1-\pi\right)\bar{p} & \pi\left( 1-\pi\right)\bar{p} \end{matrix} \right]$.

Then the assumed variance of $\hat{p}_{I}-\hat{p}_{N}$ under $H_{0}$ is $\frac{\sigma_{0}^{2}}{n}=\frac{\tilde{I}_{11}-\tilde{I}_{10}\tilde{I}_{00}^{-1}\tilde{I}_{01}}{n^{2}\pi^{2}\left( 1-\pi\right)^{2}}=\frac{1}{n\pi(1-\pi)}\left[ \pi p_{I}+\left( 1-\pi\right)p_{N} \right]\left[ 1-\left( \pi p_{I}+\left( 1-\pi\right)p_{N} \right) \right]=\frac{1}{n\pi(1-\pi)}\bar{p}(1-\bar{p})$, and the empirical variance of $\hat{p}_{I}-\hat{p}_{N}$ is $\frac{\hat{\sigma}_{0}^{2}}{n}=\frac{1}{n\hat{\pi}\left( 1-\hat{\pi} \right)}\hat{\bar{p}}\left( 1-\hat{\bar{p}} \right)=\frac{\frac{(a+b)(c+d)}{a+b+c+d}}{(a+c)(b+d)}=\frac{(a+b)(c+d)}{(a+b+c+d)(a+c)(b+d)}$.

Thus, the score test statistics is

$$T_{S}=\frac{\hat{p}_{I}-\hat{p}_{N}}{\frac{\hat{\sigma}_{0}}{\sqrt{n}}}=\frac{\left( \frac{a}{a+c}-\frac{b}{b+d} \right)\sqrt{a+b+c+d}}{\sqrt{\frac{(a+b)(c+d)}{(a+c)(b+d)}}}=\frac{\left( ad-bc \right)\sqrt{a+b+c+d}}{\sqrt{(a+c)(b+d)(a+b)(c+d)}}$$

Under $H_{1}$, the likelihood-based variance of $\hat{p}_{I}-\hat{p}_{N}$ is $\frac{\sigma_{1}^{2}}{n}=\frac{I_{11}-I_{10}I_{00}^{-1}I_{01}}{n^{2}\pi^{2}\left( 1-\pi\right)^{2}}=\frac{1}{n\pi(1-\pi)}\frac{p_{I}p_{N}(1-p_{I})(1-p_{N})}{\pi p_{I}\left( 1-p_{I} \right)+\left( 1-\pi\right)p_{N}(1-p_{N})}$.

1. **Expected cell counts**

The expected cell counts can be calculated assuming a care-seeking source population of size $N$, cumulative incidence of test-positive illness $\left( 1-e^{-\Lambda_{I}\left( \tau\right)} \right)$ over the study period $\tau$, and cumulative hazard of test-negative illness. The vaccine has effectiveness VE assuming an all-or-none model ^31^, where the vaccine provides full protection in a percentage of vaccinated individuals (corresponding to VE) and no protection for the rest. We assume vaccination is completed before the study starts and vaccine coverage among the source population is $p_{N}$. The expected cell counts ^32^ are as follows:

$E(a)={Np}_{N}(1-VE)\left[ 1-e^{-\Lambda_{I}(\tau)} \right]$,

$E\left( b \right)={Np}_{N}\Lambda_{N}(\tau)$,

$E(c)=N(1-p_{N})\left[ 1-e^{-\Lambda_{I}(\tau)} \right]$,

$$E(d)=N\left( 1-p_{N} \right)\Lambda_{N}(\tau)$$

Sequentially, the expected fraction vaccinated among positive tests can be approximated as $p_{I}\approx\frac{E\left( a \right)}{E\left( a \right)+E\left( c \right)}=\frac{p_{N}(1-VE)}{1-p_{N}\times NE}$.

1. **Sample sizes associated with the three tests**

An example of three sample sizes for 95% VE is shown in Table S2. The Wald sample size adding continuity correction has the largest sample size across all vaccine coverages.

| Vaccine coverage ($\boldsymbol{p}_{\boldsymbol{N}}$) | 10% | 30% | 50% | 70% | 90% |
| --- | --- | --- | --- | --- | --- |
| Wald sample size ($\boldsymbol{n}_{\boldsymbol{W}}$) | 228 | 74 | 45 | 37 | 54 |
| Wald sample size w. cc ($\boldsymbol{n}_{\boldsymbol{C}}$) | 277 | 92 | 58 | 51 | 80 |
| Score sample size ($\boldsymbol{n}_{\boldsymbol{S}}$) | 179 | 63 | 42 | 36 | 53 |

Table S2. Sample sizes for $VE=95\%$ of the three tests. Wald sample size w. cc: Wald sample size with continuity correction.

1. **Distribution of estimated variance of score statistics under null hypothesis**

The distribution of the score test statistics under $H_{1}$ varies with $a+c$, but that under $H_{0}$ does not (figure S3). From figure S3, we can see that variances of  $\hat{p}_{I}-\hat{p}_{N}$ of the test-negative design across different $a+c$ are similar to those of the case-control study. Therefore, the case-control estimated variance of the score can capture the test-negative estimated variance. Thus, we do not necessarily vary  $\sigma_{0}$ as a function of $a+c$ in the proposed test negative sample size.


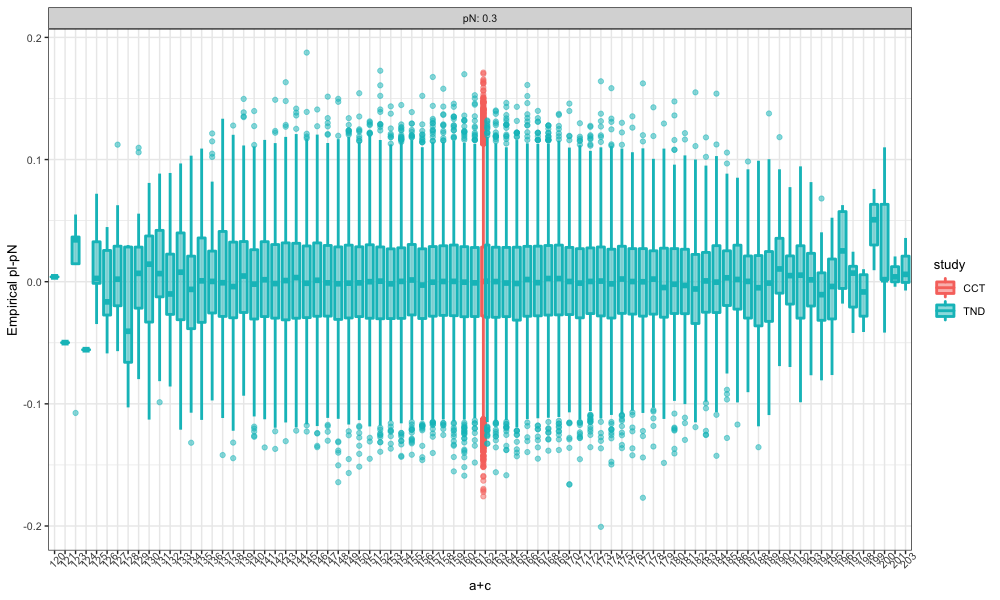


Figure S1. The distribution of the score test statistics under $H_{0}$ (VE=0) for case-control study (red) and the test-negative design (green) with various $a+c$ under 30% vaccine coverage $p_{N}$ with sample size 500.

1. **Distribution of test-negative design data**

The distribution of the test-negative design has extra variability in the column margin, compared to the case-control study, which has fixed column margin and random row margin. Thus, cell counts in the test-negative design have two-way variability, resulting in the multinomial distribution, i.e., $a,b,c,d\sim Multinomial \left( n,\pi p_{I}, \left( 1-\pi\right)p_{N}, \pi\left( 1-p_{I} \right), \left( 1-\pi\right)\left( 1-p_{N} \right) \right)$. We compared the distribution of the test-negative design cell counts (green) and the multinomial distribution random numbers (red) generated with the same parameters in figure S2. We can see that the distribution of test-negative design aligns with the multinomial distribution.


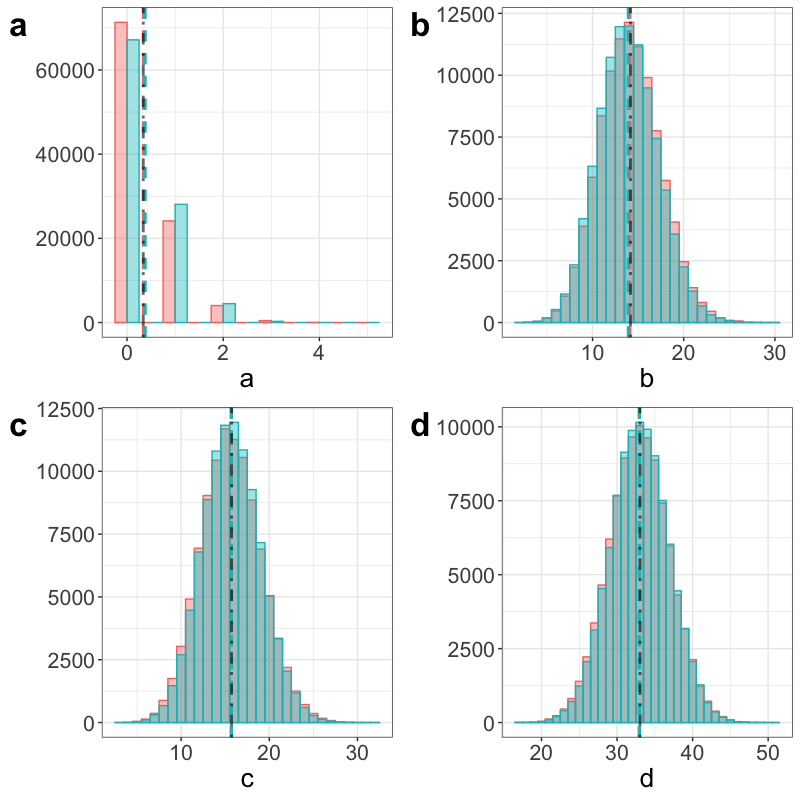


Figure S2. The distribution of test-negative design cell counts (green) and multinomial distribution random numbers (red) for 30% vaccine coverage and 95% vaccine effectiveness.

1. **Distribution-based variance and likelihood-based variance under the alternative**

The variance under the alternative adopted in the proposed sample size is derived as below.

$$\tilde{\sigma}_{1}^{2}\left( \hat{\pi} \right)=nVar\left( \hat{p}_{I}-\hat{p}_{N}|\hat{\pi} \right)=nVar\left( \frac{1}{n\hat{\pi}}\sum_{i in TP} X_{i}-\frac{1}{n(1-\hat{\pi})}\sum_{j in TN} X_{j}|\hat{\pi} \right)=n\left[ Var\left( \frac{1}{n\hat{\pi}}\sum_{i in TP} X_{i}|\hat{\pi} \right)+Var\left( \frac{1}{n(1-\hat{\pi})}\sum_{j in TN} X_{j}|\hat{\pi} \right)-2Cov\left( \frac{1}{n\hat{\pi}}\sum_{i in TP} X_{i},\frac{1}{n(1-\hat{\pi})}\sum_{j in TN} X_{j}|\hat{\pi} \right) \right]=n\left[ \frac{p_{I}\left( 1-p_{I} \right)}{n\hat{\pi}}+\frac{p_{N}\left( 1-p_{N} \right)}{n\left( 1-\hat{\pi} \right)}-2\left( -\frac{1}{n}p_{I}p_{N} \right) \right]=\frac{p_{I}\left( 1-p_{I} \right)}{\hat{\pi}}+\frac{p_{N}\left( 1-p_{N} \right)}{\left( 1-\hat{\pi} \right)}+2p_{I}p_{N}$$

On the other hand, the variance under the alternative derived from the likelihood of logistic regression varying with  $\hat{\pi}$ is $\sigma_{1}^{2}(\hat{\pi}) =\frac{1}{\hat{\pi}(1-\hat{\pi})}\frac{p_{I}p_{N}(1-p_{I})(1-p_{N})}{\hat{\pi}p_{I}\left( 1-p_{I} \right)+\left( 1-\hat{\pi} \right)p_{N}(1-p_{N})}$. To be consistent with the case-control score sample size, we were supposed to utilize likelihood-based $\sigma_{1}^{2}(\hat{\pi})$ rather than distribution-based $\tilde{\sigma}_{1}^{2}\left( \hat{\pi} \right)$. However, as shown in figure S3, the distribution-based variance (yellow) is a better estimate of the empirical variance of $\hat{p}_{I}-\hat{p}_{N}$, the simplified score, and the likelihood-based variance (purple) underestimates it for 10%, 30%, and 50% vaccine coverage. Though the distribution-based variance tends to overestimate for 70% and 90% vaccine coverage, we adopted the distribution-based variance in the proposed sample size.


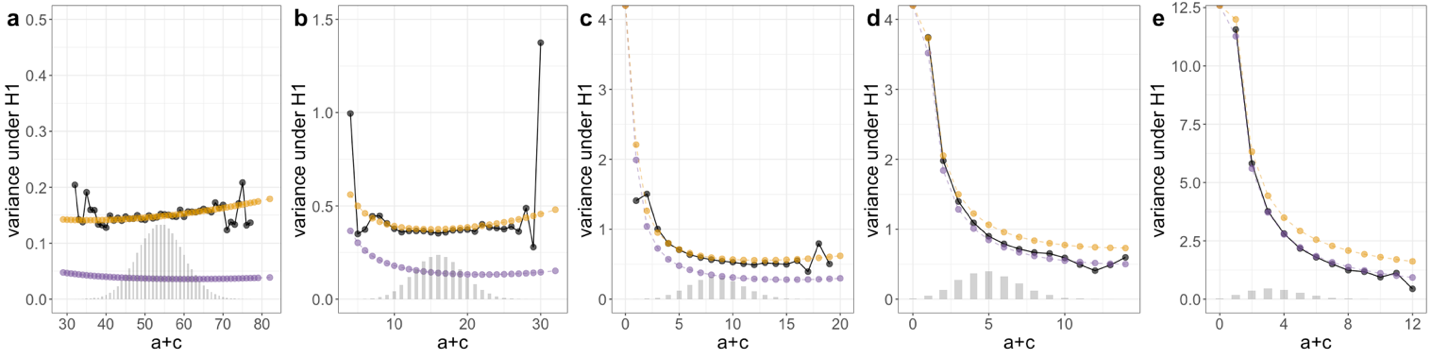


Figure S3.  Empirical variance of $\hat{p}_{I}-\hat{p}_{N}$ (black), likelihood-based$\sigma_{1}^{2}(\hat{\pi})$ (purple) and distribution-based $\tilde{\sigma}_{1}^{2}(\hat{\pi})$ (yellow) along with the distribution of test positives (histogram in grey), $a+c$, for vaccine coverage $p_{N}=$ 10% (panel a), 30% (panel b), 50% (panel c), 70% (panel d), 90% (panel e) with 95% vaccine effectiveness.

Besides, the likelihood-based variance $\sigma_{1}^{2}(\hat{\pi})$ yields insufficient sample size (Table S3) and under power for lower vaccine coverage (Table S4). There are several reasons to adopt the distribution-based variance. In the sample size formular, larger variance under the alternative yields larger sample size and conservative power. In contrast to a lower power, a conservative sample size is preferred. Moreover, the power of the TND for high vaccine effectiveness and low vaccine coverage, as shown in figure 6, needs the biggest improvement. We focus more on the lower vaccine coverage scenarios rather than higher.

| VE | Vaccine Coverage ($\boldsymbol{p}_{\boldsymbol{N}}$) | | | | | | | | | | |
| --- | --- | --- | --- | --- | --- | --- | --- | --- | --- | --- | --- |
|  | **10%** | | **30%** | | **50%** | | **70%** | | **90%** | | |
|  | $\boldsymbol{n}_{\boldsymbol{s}}$ | ${\tilde{\boldsymbol{n}}}_{\boldsymbol{p}}$ | $\boldsymbol{n}_{\boldsymbol{s}}$ | ${\tilde{\boldsymbol{n}}}_{\boldsymbol{p}}$ | $\boldsymbol{n}_{\boldsymbol{s}}$ | ${\tilde{\boldsymbol{n}}}_{\boldsymbol{p}}$ | $\boldsymbol{n}_{\boldsymbol{s}}$ | ${\tilde{\boldsymbol{n}}}_{\boldsymbol{p}}$ | $\boldsymbol{n}_{\boldsymbol{s}}$ | ${\tilde{\boldsymbol{n}}}_{\boldsymbol{p}}$ |  |
| **90%** | 225 | 228 | 81 | 84 | 55 | 59 | 50 | 55 | 82 | 89 |  |
| **95%** | 179 | 183 | 63 | 67 | 46 | 46 | 36 | 41 | 53 | 63 |  |

Table S3. TND score sample size with likelihood-based variance varying with  $\hat{\pi}$ ($\tilde{n}_{p}$) and the case-control score sample size ($n_{s}$) for 90% and 95% vaccine effectiveness (VE) across 10%-90% vaccine coverage ($p_{N}$) with 0.025 type I error and 80% desired power.

| VE | Vaccine Coverage ($\boldsymbol{p}_{\boldsymbol{N}}$) | | | | | | | | | |
| --- | --- | --- | --- | --- | --- | --- | --- | --- | --- | --- |
|  | **10%** | | **30%** | | **50%** | | **70%** | | **90%** | |
|  | $\boldsymbol{n}_{\boldsymbol{s}}$ | ${\tilde{\boldsymbol{n}}}_{\boldsymbol{p}}$ | $\boldsymbol{n}_{\boldsymbol{s}}$ | ${\tilde{\boldsymbol{n}}}_{\boldsymbol{p}}$ | $\boldsymbol{n}_{\boldsymbol{s}}$ | ${\tilde{\boldsymbol{n}}}_{\boldsymbol{p}}$ | $\boldsymbol{n}_{\boldsymbol{s}}$ | ${\tilde{\boldsymbol{n}}}_{\boldsymbol{p}}$ | $\boldsymbol{n}_{\boldsymbol{s}}$ | ${\tilde{\boldsymbol{n}}}_{\boldsymbol{p}}$ |
| **90%** | 0.76 | 0.77 | 0.75 | 0.77 | 0.78 | 0.80 | 0.78 | 0.81 | 0.79 | 0.81 |
| **95%** | 0.69 | 0.73 | 0.72 | 0.78 | 0.73 | 0.78 | 0.75 | 0.80 | 0.79 | 0.83 |

Table S4. Simulated power based on the TND score sample size with likelihood-based variance varying with  $\hat{\pi}$ ($\tilde{n}_{p}$) and the case-control score sample size ($n_{s}$) for 90% and 95% vaccine effectiveness (VE) across 10%-90% vaccine coverage ($p_{N}$) with 0.025 type I error and 80% desired power.
